# Monogeneans on exotic Indian freshwater fish. 8. Co-translocation of *Cichlidogyrus tilapiae* (Monogenea, Dactylogyridae) with pindani *Chindongo socolofi* (Cichliformes, Cichlidae): first report of this parasite genus in India within aquarium trade facilities

**DOI:** 10.1051/parasite/2025046

**Published:** 2025-07-29

**Authors:** Amit Tripathi, Chawan Matey, Antoine Pariselle, Maarten P. M. Vanhove

**Affiliations:** 1 Department of Zoology, University of Lucknow Uttar Pradesh 226 007 India; 2 Institute of Evolutionary Science of Montpellier (ISEM), Centre National de la Recherche Scientifique (CNRS), Université de Montpellier, Institut de Recherche pour le Développement (IRD) 34095 Montpellier France; 3 Laboratory Biodiversity, Ecology and Genome, Faculty of Sciences, Mohammed V University in Rabat 10000 Rabat Morocco; 4 Research Group Zoology: Biodiversity & Toxicology, Centre for Environmental Sciences, Hasselt University 3590 Diepenbeek Belgium

**Keywords:** Parasites, Ornamental fish trade, 18S-ITS1 and 28S rRNA genes, Haplotype

## Abstract

The pindani, *Chindongo socolofi* (Cichliformes, Cichlidae) is a popular freshwater ornamental fish from Lake Malawi in Africa. Although identifying parasites associated with the global ornamental fish trade is critical for developing biosecurity practices, little is known about the parasite fauna of *C. socolofi*. Therefore, this study sought to determine what monogenean parasites *C. socolofi* harbours in India. Adult specimens of this host species were collected from various aquarium shops across the country between 2020 and 2022, and their gills were subjected to parasitological examination. Monogeneans were detected in five host specimens (22.7%) with low mean intensities (6.2 ± 3.8). They were identified as *Cichlidogyrus tilapiae* (Monogenea: Dactylogyridae) based on the presence of the following morphometric characteristics: two pairs of anchors, two auricles on the dorsal bar, a V-shaped ventral bar, and an accessory piece with a folded rim and a bent bifurcated tip. The morphological identification was confirmed by the sequence analysis of the specimen’s 18S-ITS1 gene regions and 28S rRNA genes to *C. tilapiae* from *Paratilapia polleni* (Cichliformes, Cichlidae) in Madagascar. This article is the first report on a species of *Cichlidogyrus* in India, found in aquarium shops, contributing to the growing list of known freshwater monogeneans that are being distributed globally via the ornamental fish trade. Additionally, it adds a new host species (*C. socolofi*) and geographic location (India, within aquarium trade) to the existing knowledge of *C. tilapiae*, a widespread and often co-introduced tropical fish parasite.

## Abstract (fr)

Le pindani, *Chindongo socolofi* (Cichliformes, Cichlidae), est un poisson d’ornement d’eau douce populaire originaire du lac Malawi, en Afrique. Bien que l’identification des parasites associés au commerce mondial des poissons d’ornement soit essentielle au développement de pratiques de biosécurité, on sait peu de choses sur la faune parasitaire de *C. socolofi*. Par conséquent, cette étude visait à déterminer quels monogènes parasites *C. socolofi* héberge en Inde. Des spécimens adultes de cette espèce hôte ont été collectés dans divers magasins d’aquariophilie du pays entre 2020 et 2022, et leurs branchies ont été soumises à un examen parasitologique. Des monogènes ont été détectés chez cinq spécimens hôtes (22,7 %) avec de faibles intensités moyennes (6,2 ± 3,8). Ils ont été identifiés comme *Cichlidogyrus tilapiae* (Monogenea : Dactylogyridae) grâce à la présence des caractéristiques morphométriques suivantes : deux paires d’ancres, deux auricules sur la barre dorsale, une barre ventrale en V et une pièce accessoire avec un bord recourbé et une extrémité bifurquée et courbée. L’identification morphologique a été confirmée par l’analyse de la séquence des régions du gène 18S-ITS1 et des gènes de l’ARNr 28S du spécimen de *C. tilapiae* de *Paratilapia polleni* (Cichliformes, Cichlidae) à Madagascar. Cet article est le premier rapport sur la présence d’une espèce de *Cichlidogyrus* en Inde, dans des aquariums, et contribue à compléter la liste croissante des monogènes d’eau douce connus et introduits dans le monde entier via le commerce des poissons d’ornement. De plus, il ajoute une nouvelle espèce hôte (*C. socolofi*) et une nouvelle localisation géographique (l’Inde, dans des magasins d’aquariophilie) aux connaissances existantes sur *C. tilapiae*, un parasite répandu et souvent co-introduit chez les poissons tropicaux.

## Introduction

*Cichlidogyrus* Paperna, 1960 (Monogenea: Dactylogyridae) is the most species-rich African freshwater monogenean genus [37, 64]. It has 141 valid species [93] naturally parasitising primarily African cichlids (Cichlidae) and a few representatives of Cyprinodontidae Wagner, 1828 (Cyprinodontiformes) and Nandidae Bleeker, 1852 (Anabantiformes) [12, 20]. Some of these species have been identified as potentially pathogenic to fish, especially in aquaculture stocks [31, 56, 69]. *Cichlidogyrus* species, with few exceptions, are quite host-specific ([64], but also see [40]). An exception is *Cichlidogyrus tilapiae* Paperna, 1960. Since its first description from the Nile Tilapia, *Oreochromis niloticus* (Linnaeus 1758) (Cichliformes: Cichlidae) in Israel, *C. tilapiae* has been recorded in 31 different fish species in 27 countries across five continents, including Asia, Africa, North America, South America, and Australia (Table 1).

**Table 1:** Global distribution of *Cichlidogyrus tilapiae* Paperna, 1960 for 27 countries and 31 host fishes.

Country/Host fish	Reference
**Australia**	
*Oreochromis mossambicus* (Peters, 1852)	[92]
**Bangladesh**	
*Oreochromis niloticus* (Linnaeus, 1758)	[17]
*Oreochromis mossambicus* (Peters, 1852)	[17]
**Brazil**	
*Coptodon rendalli* (Boulenger, 1897)	[13]
*Oreochromis niloticus* (Linnaeus, 1758)	[10, 91]
**Burkina Faso**	
*Oreochromis niloticus* (Linnaeus, 1758)	[9]
**Burundi**	
*Oreochromis niloticus* (Linnaeus, 1758)	[29]
**Cameroon**	
*Chromidotilapia guntheri* (Sauvage, 1882)	[51]
*Chromidotilapia linkei* Staeck, 1980	[51]
*Coptodon camerunensis* (Lönnberg, 1903)	[65]
*Coptodon gutturosa* Stiassny, Schliewen and Dominey, 1992	[65]
*Pelmatolapia mariae* Boulenger, 1899	[65]
*Tilapia kottae* (Lönnberg, 1904) [now *Coptodon kottae* Lönnberg, 1904]	[65]
**China**	
*Coptodon zillii* (Gervais, 1848)	[94]
*Sarotherodon galilaeus* (Linnaeus, 1758)	[94]
*Oreochromis mossambicus* (Peters, 1852)	[94]
*Oreochromis niloticus* (Linnaeus, 1758)	[94]
**Colombia**	
*Oreochromis mossambica* (Peters, 1852) [now *Oreochromis mossambicus* (Peters, 1852)]	[35]
**Democratic Republic of the Congo**	
Coptodon tholloni (Sauvage, 1884)	[27, 29]
*Oreochromis macrochir* (Boulenger, 1912)	[29]
Oreochromis *mortimeri* (Trewavas, 1966)	[28]
*Oreochromis mweruensis* Trewavas, 1983	[28–30, 32]
*Oreochromis niloticus* (Linnaeus, 1758)	[29, 30]
**Côte d’Ivoire**	
*Oreochromis niloticus* (Linnaeus, 1758)	[5]
**Cuba**	
*Oreochromis aureus* (Steindachner, 1864)	[49, 68]
**Egypt**	
*Oreochromis niloticus* (Linnaeus, 1758)	[4, 15]
*Coptodon zillii* (Gervais, 1848)	[15]
**Ghana**	
*Oreochromis niloticus* (Linnaeus, 1758)	[58, 59, 62]
*Sarotherodon galilaeus* (Linnaeus, 1758)	[58, 59, 62]
*Tilapia busumana* (Günther, 1903)	[59]
*Hemichromis fasciatus* (Peters, 1857)	[59]
*Chromidotilapia guntheri* (Sauvage, 1882)	[60, 62]
*Oreochromis aureus* (Steindachner, 1864)	[62]
**Israel**	
*Oreochromis niloticus* (Linnaeus, 1758)	[57]
*Sarotherodon galilaeus* (Linnaeus, 1758)	[57, 58]
*Tristramella sacra* (Günther, 1865)	[57]
*Tristramella simonis* (Günther, 1864)	[57]
**Japan**	
*Oreochromis mossambicus* (Peters, 1852)	[44]
*Oreochromis niloticus niloticus* (Linnaeus, 1758)	[44]
**Kenya**	
*Oreochromis niloticus* (Linnaeus, 1758)	[71]
*Oreochromis leucostictus* (Trewavas, 1933)	[71]
**Madagascar**	
*Oreochromis mossambicus* (Peters, 1852)	[77]
*Oreochromis niloticus* (Linnaeus, 1758)	[77]
*Coptodon rendalli* (Boulenger, 1897)	[77]
*Pachypanchax omalonotus* (Duméril, 1861)	[77]
*Ptychochromis oligacanthus* (Bleeker, 1868)	[77]
*Paratilapia polleni* Bleeker, 1868	[77]
*Paretroplus polyactis* Bleeker, 1878	[77]
**Malaysia**	
*Oreochromis niloticus* (Linnaeus, 1758)	[41]
Red hybrid tilapia (*Oreochromis mossambicus* × *O. niloticus*)	[41]
**Mexico**	
*Oreochromis aureus* (Steindachner. 1864)	[26]
*Oreochromis niloticus* (Linnaeus, 1758)	[26, 50, 63]
*Vieja fenestrata* (Günther, 1860)	[26]
**Nigeria**	
*Chromidotilapia guntheri* (Sauvage, 1882)	[51]
**Philippines**	
*Oreochromis niloticus* (Linnaeus, 1758)	[2, 52]
**Senegal**	
*Hemichromis fasciatus* Peters, 1857	[48]
*Oreochromis niloticus* (Linnaeus, 1758)	[48]
*Sarotherodon galilaeus* (Linnaeus, 1758)	[48]
*Coptodon guineensis* Günther, 1862	[48]
**South Africa**	
*Oreochromis mossambicus* (Peters, 1852)	[42, 43, 54]
*Pseudocrenilabrus philander* (Weber, 1897)	[54]
**Tanzania**	
*Oreochromis urolepis* (Norman, 1922)	[62]
**Thailand**	
*Oreochromis niloticus* (Linnaeus, 1758)	[45]
*O. niloticus x O. mossambicus*	[45]
**Uganda**	
*Oreochromis niloticus* (Linnaeus, 1758)	[62]
*Oreochromis spilurus* (Günther, 1894)	[62]
*Oreochromis leucostictus* (Trewavas 1933)	[61]
*Haplochromis macrognathus* Regan, 1922	[61]
*Oreochromis mossambicus* (Peters, 1852)	[61]
**Zimbabwe**	
*Oreochromis mortimeri* (Trewavas, 1966)	[14]

Pindani, *Chindongo* (*Pseudotropheus*) *socolofi* (Johnson, 1974) (Cichliformes, Cichlidae) is native to Lake Malawi in Africa [18, 46], and is available in two colour variants – normal (Blue pindani) and albino (White pindani) [33]. Despite the importance of *C*. *socolofi* in the ornamental fish market [74], little is known about its parasite fauna. To our knowledge, only one study on parasitic infections of *C*. *socolofi* exists [11]. These researchers studied the parasites of cichlids imported via the aquarium trade in Türkiye and recorded the protozoan parasite *Trichodina pediculus* Ehrenberg, 1831.

This study aimed to establish whether *C*. *socolofi* is infected by monogenean parasites and, if so, whether they were co-translocated into India via the ornamental fish trade. We demonstrate the presence of *C*. *tilapiae* in post-quarantine populations of *C*. *socolofi* collected from Indian aquarium markets. This was accomplished first by morphological characterisation (structure and measurements of the sclerotised parts of the haptor and reproductive organs) and subsequently by molecular characterisation (Sanger sequencing of 18S rRNA gene-ITS1 region and 28S rRNA genes). This paper is part of a series on exotic and/or invasive monogenean parasites imported into India via the ornamental trade [81–87].

## Materials and methods

### Ethics

This study was approved by the institutional ethics committee of the University of Lucknow under the protocol numbers LU/AEC/ZOO/2019 and 19/I/2024/IAEC/LU.

### Sample collection and examination

**Figure 1:**
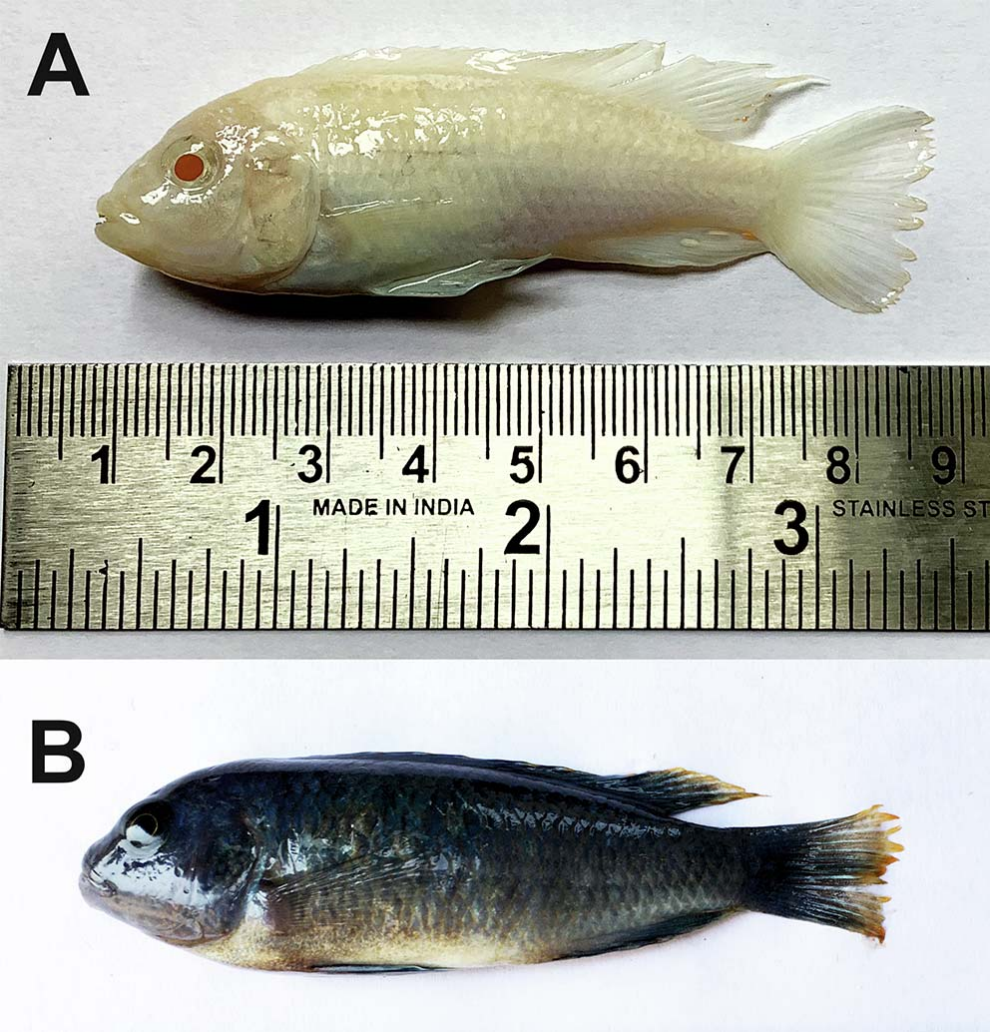
Freshly dead specimens of *Chindongo socolofi* (Johnson, 1974) examined for the present study. A. Blue pindani, B. White pindani. Photograph by Chawan Matey.

Between January 2020 and December 2022, 22 specimens of *C*. *socolofi* (total weight: 3.12–6.50 g; and total length: 4.5–8.0 cm) (Fig. 1) were collected from aquarium shops in Lucknow, New Delhi, and Kolkata, India. Fish were shipped to the laboratory the same day after they were packaged in polybags containing water and pure oxygen. Individual fish were euthanised with an overdose of tricaine methanesulfonate (MS-222 @ 150 mg/L; Sigma Aldrich Co., St. Louis, MO, USA), followed by exsanguination by the removal of gill arches. Half of the gill arches were initially fixed in hot (60 °C) distilled water to relax and heat-kill the specimens before they were transferred to 4% formalin for microscopy following Kritsky [36]. The other half was preserved in 95% ethanol for genetic analysis. Some of the gill arches were examined fresh with live worms. Monogeneans were later isolated from these gills using fine dissecting needles under a stereomicroscope (Leica Microsystems, Wetzlar, Germany). Fish specimens were identified morphologically with the help of the ICAR-National Bureau of Fish Genetic Resources (ICAR-NBFGR), a premier Indian institute on fish taxonomy, biology, and genomics.

### Morphological analysis

Formalin-fixed worms were stained with either Gomori’s trichrome or Borax carmine and mounted in DPX (dibutylphthalate polystyrene xylene) for observing internal anatomy (permanent mounts); others were mounted in glycerine jelly or Hoyer’s medium for the study of sclerotised parts of the haptor and reproductive organs (temporary mounts). Additionally, some ethanol-preserved worms were treated for 20–30 min at 55 °C with 1.0 μL of digestion buffer (0.1 μl of solid tissue buffer and 0.9 μL proteinase *K*) (Quick DNA^TM^ Miniprep Plus Kit, ZYMO Research, Irvine, CA, USA) to digest the tissues surrounding their sclerotised parts.

The morphology of the sclerotised parts was examined under a light microscope (Leica DM4B) at a magnification of 100×, using an oil immersion lens with phase-contrast (PHA-CO) and differential interference contrast illumination. Photographs and measurements (in micrometres) were obtained using a digital camera (Leica DFC7000 T) and imaging analysis software (LAS X; Leica Microsystems Ltd.) attached to the light microscope. A composite line drawing plate was made from multiple parasite specimens using an Olympus BX-51 microscope drawing tube. Species were identified based on the morphological characters described in previous studies [14, 15, 35, 44, 57, 64]. The terminology and measurement of these characters followed Rahmouni *et al*. [70]. The prevalence and mean intensity of infection were calculated according to Bush et al. [8].

### DNA extraction and amplification

Representative samples of ethanol-preserved specimens were morphologically identified as conspecific to the temporary and permanent mounts before being pooled in two groups (*n* = 3) by two collection sites (Lucknow and New Delhi) for gDNA isolation using a DNA extraction kit (Extracta DNA Prep for PCR-Tissue, Quantabio, Beverly, MA, USA), according to the manufacturer’s instructions. Partial fragments of 18S ribosomal RNA genes (18S) and internal transcribed spacer 1 (ITS1) clusters were amplified with the primers s1 [78] and ir8 [75]. Meanwhile, those of 28S ribosomal RNA genes were amplified with the primers c1 and d2 [23].

Polymerase chain reactions were performed in an automated thermal cycler (Himedia Laboratories, Thane, MH, India) with reaction mixtures (final volume 20 μL) containing 4 μL of distilled water, 10 μL of 2× PCR TaqMixture (Himedia Laboratories), 1 μL of 10 pmol/microliter of each primer, and 4 μL of DNA template. The amplification profile for the 18S rRNA gene-ITS1 region was as follows: initial denaturation at 95 °C for 3 min, then 35 cycles of denaturation at 95 °C for 30 s, annealing at 50 °C for 30 s, and extension at 72 °C for 1 min, with a final extension at 72 °C for 7 min. The amplification profile for the 28S ribosomal RNA gene followed Šimková *et al*. [76]. The size of the PCR products (2 μL) was analysed by electrophoresis in 1.2% agarose gel prepared in 1× TAE buffer, prestained with 0.1 μL/mL 10,000× Sybr Safe in dimethyl sulfoxide (Invitrogen, Waltham, MA, USA), at 90 V for 30 min, and visualised and documented on a Bio-Print gel documentation imaging system (Vilber Lourmat, Collégien, France).

### Sequence analysis

The PCR products were purified (on 1.5% agarose using a QIAquick PCR Purification Kit; QIAGEN, Germantown, MA, USA) and Sanger sequenced (on an ABI 3730xL automated sequencer; Applied Biosystems, Foster City, CA, USA) with PCR primers by Eurofins Genomics (Bengaluru, KA, India). SnapGene version 5.3 (https://www.snapgene.com) was used to manually quality-trim the successfully sequenced amplicons. Consensus sequences (18S-ITS1, 942 bp; 28S, 660 bp and 848 bp) were generated using the BioEdit Program [22].

Sequences, together with all sequences from the same markers and species retrieved from NCBI GenBank (Tables 2 and 3) were aligned using ClustalW [24] implemented in MEGA v.7 [38]. To obtain equal lengths for sequence analysis, they were trimmed to 687 bp (18S-ITS1) and 631 bp (28S). A median-joining network [3] was inferred for each marker using PopART [39].

**Table 2:** Information on *Cichlidogyrus tilapiae* Paperna, 1960, including hosts, localities, and GenBank accession numbers of their 18S+ITS1 rRNA gene sequences (as retrieved from the NCBI database on December 04, 2024).

Isolates	Host fish	Geographic location	Accession No.	Reference
AT.CT-2021	*Chindongo socolofi*	India, Asia	MZ266637	This study
PPZIM199_1	*Coptodon rendalli*	Zimbabwe, Southern Africa	ON819336	[21]
PPZIM200_1	*Coptodon rendalli*	Zimbabwe, Southern Africa	ON819337	[21]
PPZIM200_2	*Coptodon rendalli*	Zimbabwe, Southern Africa	ON819338	[21]
PPZIM200_3	*Coptodon rendalli*	Zimbabwe, Southern Africa	ON819339	[21]
PPZIM200_4	*Coptodon rendalli*	Zimbabwe, Southern Africa	ON819340	[21]
PPZIM056_1	*Oreochromis niloticus*	Zimbabwe, Southern Africa	ON819297	[21]
PPZIM059_1	*Oreochromis niloticus*	Zimbabwe, Southern Africa	ON819298	[21]
PPZIM105_1	*Oreochromis niloticus*	Zimbabwe, Southern Africa	ON819310	[21]
PPZIM117_1	*Oreochromis niloticus*	Zimbabwe, Southern Africa	ON819312	[21]
PPZIM174_1	*Oreochromis cf. mortimeri*	Zimbabwe, Southern Africa	ON819331	[21]
PPKAT465_1	*Oreochromis niloticus*	D. R. Congo, Central Africa	ON819262	[21]
PPKAT482_1	*Oreochromis niloticus*	D. R. Congo, Central Africa	ON819266	[21]
PPKAT485_2	*Oreochromis niloticus*	D. R. Congo, Central Africa	ON819268	[21]
PPKAT495_1	*Oreochromis niloticus*	D. R. Congo, Central Africa	ON819269	[21]
PPKAT997_1	*Oreochromis mweruensis*	D. R. Congo, Central Africa	ON819289	[21]
PPKAT1074_2	*Coptodon rendalli*	D. R. Congo, Central Africa	ON819246	[21]
PPKAT1074_3	*Coptodon rendalli*	D. R. Congo, Central Africa	ON819247	[21]
PPKAT1074_4	*Coptodon rendalli*	D. R. Congo, Central Africa	ON819248	[21]
PPKAT1039_1	*Oreochromis aureus*	D. R. Congo, Central Africa	ON819235	[21]
PPKAT1039_2	*Oreochromis aureus*	D. R. Congo, Central Africa	ON819236	[21]
PPKAT1039_3	*Oreochromis aureus*	D. R. Congo, Central Africa	ON819237	[21]
PPKAT1039_5	*Oreochromis aureus*	D. R. Congo, Central Africa	ON819238	[21]
PPKAT1002_1	*Oreochromis mweruensis*	D. R. Congo, Central Africa	ON819220	[21]
PPCAM347_2	*Coptodon guineensis*	D. R. Congo, Central Africa	ON819204	[21]
PPCAM334_1	*Coptodon guineensis*	D. R. Congo, Central Africa	ON819198	[21]
PPCAM339_1	*Coptodon guineensis*	D. R. Congo, Central Africa	ON819199	[21]
PPCAM340_1	*Coptodon guineensis*	D. R. Congo, Central Africa	ON819200	[21]
PPCAM340_2	*Coptodon guineensis*	D. R. Congo, Central Africa	ON819201	[21]
PPCAM342_1	*Coptodon guineensis*	D. R. Congo, Central Africa	ON819202	[21]
PPCAM347_1	*Coptodon guineensis*	D. R. Congo, Central Africa	ON819203	[21]
PPCAM037_1	*Oreochromis niloticus*	Cameroon, Central Africa	ON819182	[21]
PPCAM059_1	*Oreochromis niloticus*	Cameroon, Central Africa	ON819186	[21]
PPCAM083_1	*Oreochromis niloticus*	Cameroon, Central Africa	ON819187	[21]
PPCAM088_2	*Oreochromis niloticus*	Cameroon, Central Africa	ON819189	[21]
RAKAN10	–	Egypt, North Africa	OR793160	Unpublished
KMC43	*Oreochromis mossambicus*	Madagascar, East Africa	MH767396	[77]
KMC44	*Oreochromis niloticus*	Madagascar, East Africa	MH767397	[77]
KMC45	*Pachypanchax omalonotus*	Madagascar, East Africa	MH767398	[77]
KMC46	*Ptychochromis oligacanthus*	Madagascar, East Africa	MH767399	[77]
KMC47	*Paratilapia polleni*	Madagascar, East Africa	MH767400	[77]
PC43	*Hemichromis fasciatus*	Senegal, West Africa	HE792797	[48]
1	*Sarotherodon galilaeus*	Côte d’Ivoire, West Africa	AJ920276	[66]
2	*Sarotherodon galilaeus*	Côte d’Ivoire, West Africa	AJ920277	[66]
3	*Sarotherodon galilaeus*	Côte d’Ivoire, West Africa	AJ920275	[66]

**Table 3:** Information on *Cichlidogyrus tilapiae* Paperna, 1960, including hosts, localities, and GenBank accession numbers of their 28S rRNA gene sequences (as retrieved from the NCBI database on December 04, 2024).

Isolates	Host fish	Geographic location	Accession No.	Reference
AT-2021	*Chindongo socolofi*	India, Asia	MZ265190	This study
CT-28S-CS-NDLS-22	*Chindongo socolofi*	India, Asia	PQ675652	This study
B2	*Oreochromis mossambicus*	China, Asia	PP448734	Unpublished
e2-7	*Oreochromis niloticus*	China, Asia	OR557581	Unpublished
E3-3	*Oreochromis niloticus*	China, Asia	OR488785	Unpublished
208CtilOnilMonzi	*Oreochromis niloticus*	D. R. Congo, Central Africa	OM720051	[30]
217CtilOnilMonzi	*Oreochromis niloticus*	D. R. Congo, Central Africa	OM720052	[30]
238CtilOnilNdim	*Oreochromis niloticus*	D. R. Congo, Central Africa	OM720053	[30]
64CtilOmwKipo	*Oreochromis mweruensis*	D. R. Congo, Central Africa	OM720054	[30]
KMC59	*Paratilapia polleni*	Madagascar, East Africa	MH767412	[77]
KMC58	*Ptychochromis oligacanthus*	Madagascar, East Africa	MH767411	[77]
KMC57	*Pachypanchax omalonotus*	Madagascar, East Africa	MH767410	[77]
KMC56	*Oreochromis niloticus*	Madagascar, East Africa	MH767409	[77]
KMC55	*Oreochromis mossambicus*	Madagascar, East Africa	MH767408	[77]
KMC54	*Coptodon rendalli*	Madagascar, East Africa	MH767407	[77]
–	*Hemichromis fasciatus*	Senegal, West Africa	HQ010029	[47]

As additional assessment for species-level identification, intraspecific genetic differences (also known as genetic distances) among different geographical isolates of *C*. *tilapiae* were computed from the same dataset. This was done using the Kimura two-parameter (K2P) model [34] of nucleotide substitution in MEGA 11 [79], with gaps treated as complete deletions.

## Results

Thirty individuals of a single monogenean species – namely *Cichlidogyrus tilapiae* Paperna, 1960 – were collected from the gills of five specimens of *C. socolofi*, with a low prevalence (22.72%) and low infection intensities (7–13 worms/fish). Brief morphological and molecular data of this species are presented below.

### Taxonomic summary

Phylum Platyhelminthes Minot, 1876

Superclass Neodermata Ehlers, 1985

Class Monogenea van Beneden, 1858

Family Dactylogyridae Yamaguti, 1963

Genus *Cichlidogyrus* Paperna, 1960

### *Cichlidogyrus tilapiae* Paperna, 1960 (Figs. 2 and 3)

Type host and locality: Nile tilapia *Oreochromis niloticus* (Linnaeus, 1758) (Cichliformes: Cichlidae); Israel (Paperna 1960).

**Figure 2:**
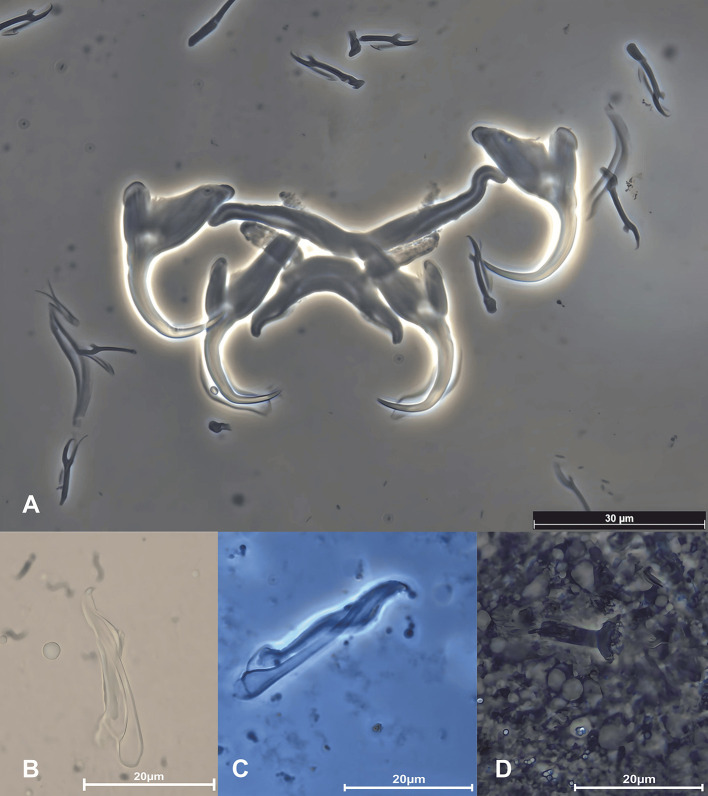
Photomontage of light microscopy and phase contrast (PHACO) images of *Cichlidogyrus tilapiae* Paperna, 1960 from *Chindongo socolofi* (Johnson, 1974). A. anchor-bar complex and hooks, B and C. different configurations of male copulatory organ, D. vagina. Photograph by Amit Tripathi.

**Figure 3:**
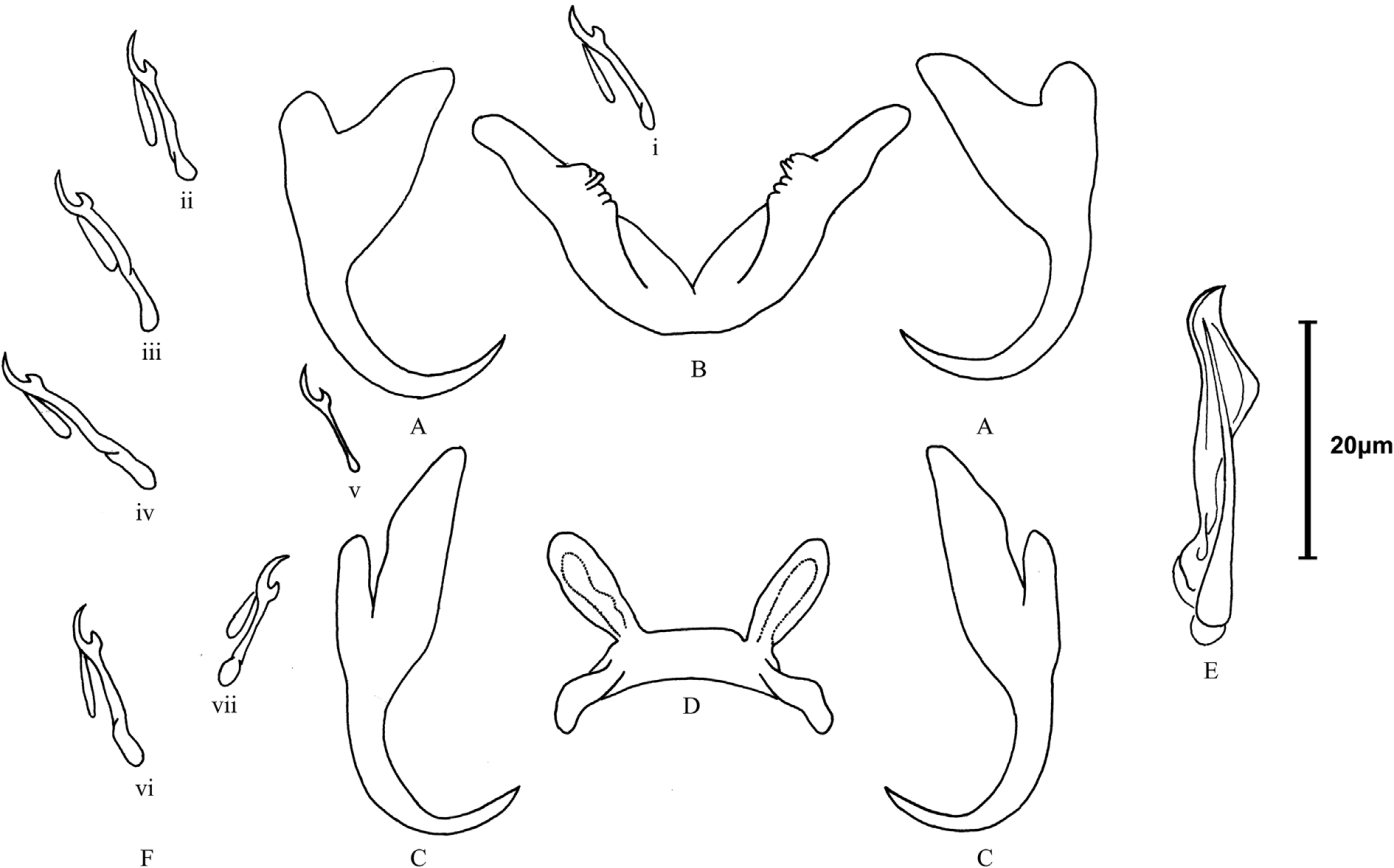
Line drawings of sclerotised structures of *Cichlidogyrus tilapiae* Paperna, 1960 from *Chindongo socolofi* (Johnson, 1974)*.* A. ventral anchor, B. ventral bar, C. dorsal anchor, D. dorsal bar, E. male copulatory organ, F. hook (pairs i–vii). Scale bar = 20 μm. Figure by Amit Tripathi.

Present host: *Chindongo socolofi* (Cichliformes, Cichlidae).

Present material and collection date: Aquarium shops in Lucknow (26.8467° N, 80.9462° E), New Delhi (28.6139° N, 77.2090° E), and Kolkata (22.5726° N, 88.3639° E), India; January 2020–December 2022.

Site of infection: Gills.

Infection parameters: Prevalence: 22.72% (5 out of 22 *C*. *socolofi* examined); Mean infection intensity: 6.2 ± 3.86 (7–13; *n* = 5).

Museum material: Five voucher specimens stained with Gomori’s trichrome or Borax carmine and mounted on glass slides in DPX (Smithsonian Institution, USA; USNM 1757684-1757688).

GenBank deposition: 18S-ITS1: 942 bp (MZ266637); 28S: 660 bp (MZ265190), 848 bp (PQ675652).

### Morphological data

*Cichlidogyrus* is distinguished by a vas deferens that does not encircle the intestinal caecum, two pairs of anchors (one dorsal and one ventral), two transversal bars (a dorsal bar with two typical auricles and a V-shaped ventral bar), seven pairs of hooks, a sclerotised or non-sclerotised vagina and a sclerotised male copulatory organ comprising a male copulatory tube and (often but not always) an accessory piece [57, 88].

Our specimens presented nearly identical morphological features of sclerotised parts (both haptoral and reproductive ones) indicated in the original description [55] and subsequent redescriptions or illustrated records [14, 15, 35, 44] of *C*. *tilapiae* (Figs. 2 and 3) (Table 4). Only two minor discrepancies were observed in the morphometry of the haptoral armaments. First, our specimens had a slightly longer and deeper outer root of the dorsal anchor (4**–**6 μm), compared to their conspecifics. Second, variations were observed in the ranges of measurements of the ventral bar. Paperna [57], for example, measured the length of the ventral bar to be 34–98 μm, whereas Douëllou [14] measured it to be 26–33 μm (as we did), and Kritsky and Thatcher [35], Ergens [15], and Maneepitaksanti and Nagasawa [44] measured it to be 50–65 μm.

**Table 4:** Comparative measurements (in μm) of reproductive organs and haptoral armaments of *Cichlidogyrus tilapiae* Paperna, 1960 from India (present study) and other geographical locations.

Measurement	Paperna [57]	Kritsky & Thatcher [35]	Ergens [15]	Douëllou [14]	Maneepitaksanti & Nagasawa [44]	Present study
Country	Israel	Colombia	Egypt	Zimbabwe	Japan	India
Reproductive organ						
Copulatory tube	19**–**37	29	30**–**33	32 (30**–**36)	29 (28**–**30)	32 (25**–**35)
Accessory piece	22**–**33	31	–	33 (31**–**33)	33 (31**–**37)	31 (30**–**32)
Haptoral parts						
Dorsal anchor length	26**–**40	40	37**–**41	42 (41**–**44)	36 (35**–**38)	37 (32**–**42)
Dorsal bar length	18**–**38	28	27**–**29	29 (28**–**30)	14 (13**–**15)	28 (22**–**34)
Dorsal bar width	–	–	–	–	–	(4**–**5)
Dorsal anchor inner root	11**–**15	–	10**–**13	18 (16**–**19)	–	16 (14**–**18)
Dorsal anchor outer root	4**–**7	–	3**–**6	4 (3**–**5)	–	5 (4**–**7)
Ventral anchor length	26**–**33	31	29**–**33	34 (32**–**36)	30 (28**–**32)	33 (31**–**36)
Ventral bar length	34**–**98	57	56**–**65	32 (31**–**33)	54 (50**–**56)	29 (26**–**32)
Ventral bar width	–	–	–	–	–	5 (4**–**6)
Ventral anchor inner root	18	–	14**–**18	12 (10**–**14)	–	15 (13**–**18)
Ventral anchor outer root	4**–**7	–	3**–**5	4 (3**–**5)	–	6 (5**–**7)
Auricle length	9**–**19	–	14**–**18	–	–	12 (11**–**13)
Hook length	–	15	–	–	–	11**–**19
Pair I	12 (7**–**17)	–	18 (17**–**19)	14 (13**–**14)	13 (12**–**15)	(14**–**15)
Pair II	15 (13**–**17)	–	11(10**–**11)	11 (9**–**12)	10 (8**–**12)	14 (13**–**15)
Pair III	16 (13**–**20)	–	18 (17**–**19)	15 (13**–**17)	14 (12**–**17)	17 (16**–**18)
Pair IV	16 (13**–**20)	–	18 (17**–**19)	17 (16**–**17)	14 (12**–**15)	(18**–**19)
Pair V	13 (11**–**15)	–	18 (17**–**19)	18 (16**–**19)	16 (15**–**17)	(11**–**12)
Pair VI	15	–	18 (17**–**19)	17 (17**–**18)	16 (15**–**18)	(17**–**18)
Pair VII	21	–	18 (17**–**19)	15 (14**–**16)	14 (13**–**15)	15

– shows that these measurement values were not provided by the respective authors.

We were also able to locate the vagina in a single live specimen, which had gone unnoticed in previous studies on *C*. *tilapiae*. It resembled a short unsclerotised (muscular?) tube with a funnel-like opening at one end (Fig. 3D). We lost it quickly however, when the vitellaria burst out of the parasite body, killing it. Therefore, our identification of the vagina may not be conclusive and should be reconfirmed. *Cichlidogyrus tilapiae* has previously been adequately described/redescribed and, thus, does not need to be formally redescribed here.

### Molecular data

The partial 18S rRNA gene-ITS1 region (942 bp) and 28S rRNA genes (660 bp and 848 bp) were sequenced from two pools of *C*. *tilapiae* specimens collected from *C*. *socolofi* in aquarium shops in India. Comparative analysis of these sequences against the NCBI GenBank non-redundant database using the “megablast” algorithm (https://blast.ncbi.nlm.nih.gov/Blast.cgi) revealed “near perfect” matches for 28S rRNA (659/660 bp; 99.85% similar identity with a query coverage of 100%) and 18S rRNA-ITS1 (935/938 bp; 99.68% similar identity with a query coverage of 99%) to *C*. *tilapiae* from *Paratilapia polleni* Bleeker, 1868 (Cichliformes, Cichlidae) in Madagascar deposited in GenBank under the accession numbers MH767412 (28S) and MH767400 (18S-ITS1), respectively [77]. These findings suggest their conspecificity (Tables 5 and 6).

**Table 5:** Intraspecific genetic distances (Kimura 2-parameter model with partial deletion option) and variations between our samples and conspecific references (most similar BLAST hits) of *Cichlidogyrus tilapiae* Paperna, 1960 based on 18S rRNA gene-ITS1 region.

Sample reference (Host; Geographic location)	Identity (%)	E-value	Genetic distance		Conspecific references (Host; Geographic location)
			18S	ITS1	
*Cichlidogyrus tilapiae* MZ266637 (*Chindongo socolofi*; India)	99.68	0.00	0.000	0.000	*Cichlidogyrus tilapiae* MH767400
					(*Paratilapia polleni*; Madagascar, East Africa)
	99.78	0.00	0.000	0.002	*Cichlidogyrus tilapiae* HE792797
					(*Hemichromis fasciatus*; Senegal, West Africa)
	99.68	0.00	0.000	0.000	*Cichlidogyrus tilapiae* MH767399
					(*Oreochromis niloticus*; Madagascar, East Africa)

**Table 6:** Intraspecific genetic distances (Kimura 2-parameter model with partial deletion option) and variations between our samples and conspecific references (most similar BLAST hits) of *Cichlidogyrus tilapiae* Paperna, 1960 based on 28S rRNA gene.

Sample reference (Host; Geographic location)	Identity (%)	E-value	Genetic distance	Conspecific references (Host; Geographic location)
*Cichlidogyrus tilapiae* MZ265190 (*Chindongo socolofi*; India)	99.85	0.00	0.000	*Cichlidogyrus tilapiae* MH767412
				(*Paratilapia polleni*; Madagascar, East Africa)
	99.85	0.00	0.000	*Cichlidogyrus tilapiae* HQ010029
				(*Hemichromis fasciatus*; Senegal, West Africa)
	99.85	0.00	0.000	*Cichlidogyrus tilapiae* MH767409
				(*Oreochromis niloticus*; Madagascar, East Africa)

The intraspecific genetic distances for 28S rRNA and 18S rRNA genes between four geographic isolates of *C. tilapiae* from different hosts and geographical locations were determined at 0%, indicating their conspecificity (Tables 5 and 6). The genetic distance for the ITS1 sequence, another marker with higher variability, was also determined between 0 and 0.002% (Table 5).

**Figure 4:**
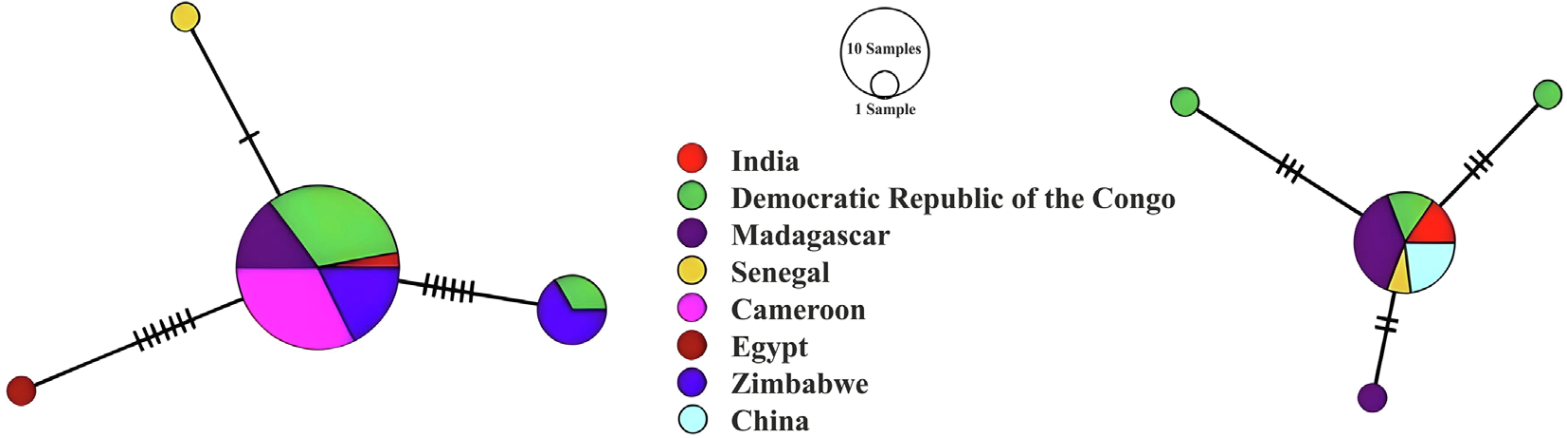
Median-joining haplotype networks based on a 687 bp fragment of small subunit rDNA and the first Internal Transcribed Spacer (left) and 631 bp of large subunit rDNA (right) from the newly sequenced individuals of *Cichlidogyrus tilapiae* from India, aligned with all previously published sequences from this species. Genotypes are represented by circles, with the size of the circle correlating with the number of isolates displaying the respective genotype. Colours denote the countries of sampling localities. Genotypes are connected by lines indicating the number of mutations between them.

The haplotype network indicated that Indian haplotypes, for both markers, were widespread, and shared with conspecifics from both native and introduced populations (Fig. 4).

## Discussion

This paper is the first to document the presence of a member of *Cichlidogyrus* in India. In addition, *C. socolofi* has been identified as a new host species for *C. tilapiae*. This is also the first time a species of *Cichlidogyrus* is formally reported from a Malawi cichlid; members of the genus are known to occur on the lake’s cichlids but were mentioned without species-level identification [6]. Although there are a few variations in morphometrical data between different geographic isolates of *C*. *tilapiae*, we do not consider these differences to merit species-level separation. These variations may be attributable to differences in the host species [55], environmental factors [7], developmental stages [85, 90], individual variations within the species, or even the different fixation [16] or measuring methods employed thus far. For instance, just as we did, Douëllou [14] measured only one branch of the ventral bar following established norms of measurements for *Cichlidogyrus* [70]. Meanwhile, Kritsky and Thatcher [35] and Ergens [15] measured the total length in a “straight line extending between the two most distant parts”. Unfortunately, Paperna [57] and Maneepitaksanti and Nagasawa [44] did not specify their measurement methods.

While many publications have recorded only the occurrence of *C. tilapiae* without providing any morphometric data, those that have provided such data have shown a few variations in the sclerotised parts. For example, Paperna [57] found that both pairs of anchors were “about the same length”. However, all subsequent investigations have clearly shown that dorsal anchors are slightly larger than the ventral anchors. Paperna [57] also described and illustrated an accessory piece that terminated in a “bent bifurcated tip”, but this bifurcation has not been observed in any other studies. Furthermore, Ergens [15] and Douëllou [14] noted a small “groove on the base of dorsal anchor”, that no other researchers have reported. Ergens [15] also illustrated a small sliver-like structure on the outer roots of the ventral anchor, which has not been described or illustrated by other researchers.

Nonetheless, the distinctive morphology of the male copulatory organ, which lacks a heel and has a hook-shaped terminal end of the accessory piece, is consistent enough in all illustrations of *C. tilapiae* to be considered the most reliable diagnostic trait for identifying this species. This aligns with the notion that identification of *Cichlidogyrus* species is primarily based on the morphology of the reproductive hard parts [89].

Curiously, the 18S rRNA gene-ITS1 region and 28S rRNA gene sequences of *C. tilapiae* found in India differed from their conspecific references by only 3 bp and 1 bp, respectively (see above). Different phenotypes of *C. tilapiae* did not cluster monophyletically in the recent morphology-based phylogeny [51]. Therefore, we speculate that either *C*. *tilapiae* comprises a species complex of morphologically variable but closely related lineages [66] or that there are geographical variants of a single species.

The haplotype networks (Fig. 4) indicate that the haplotype of *C. tilapiae* found in India occurs widely throughout native and introduced host and parasite populations. Other markers than the ones used here, for example a sequence fragment of the cytochrome *c* oxidase subunit 1 gene, allow higher resolution distinction between populations of *C. tilapiae* [30] and may allow the identification of native and (co-)introduced strains of cichlid parasites [21].

### Indian scenario

Since nothing is known about the monogenean fauna of *C. socolofi* in the wild, we cannot ascertain whether it is a natural host of *C. tilapiae* or whether it acquired it from other cichlids cohabiting in aquarium conditions. However, it is highly likely that *C. socolofi* is a regular host for *C. tilapiae* because the latter was consistently recovered over space (Lucknow, New Delhi, and Kolkata) and time (January 2020–December 2022). *Chindongo socolofi* is currently maintaining its self-sustaining populations in the country’s aquacultural ponds and has yet to be recorded in the wild. The potential negative impact of *C*. *socolofi* on India’s environment and/or economy will therefore depend on its ability to successfully establish, dominate, and expand in Indian waters. It has previously been hypothesised that the invasion success of a fish is linked to, amongst other things, favourable environmental conditions in the new habitat that are comparable to those in its native ranges (climate match theory; [1, 25]), and to the enemy release hypothesis [80]. *Chindongo socolofi* may be considered a potentially invasive fish species in this context because the climatic conditions in India, particularly in South India, are similar to those found in the native range of *C*. *socolofi* (southeastern Africa), including a tropical climate and a temperature range of 24–26 °C [18].

Should *C*. *socolofi* become invasive in Indian waters, the low host specificity of *C. tilapiae* (see above), combined with the native fish species’ lack of protective immunity against exotic parasites [72], could pose a serious biological invasion challenge. It is worth noting that *C*. *tilapiae* has already demonstrated its ability to switch from introduced cichlids to native hosts in destination environments, such as *Vieja fenestrata* (Günther, 1860) (Cichliformes, Cichlidae) (syn. = *Paraneetroplus fenestratus*) in Mexico [19], and *Coptodon tholloni* (Sauvage 1884) (Cichliformes, Cichlidae) in the Lower Congo Basin [29], and even non-cichlid hosts: *Pachypanchax omalonotus* (Duméril, 1861) (Cyprinodontiformes: Aplocheilidae) in Madagascar [77]. In fact, tilapia-infecting monogeneans have been proposed as the most ubiquitous tropical freshwater fish parasites globally, with *C. tilapiae* being one of the species most frequently reported as co-introduced with translocated tilapias [73]. However, to the best of our knowledge, this is the first report of this parasite from the ornamental fish trade.

The presence of *C. tilapiae* on *C*. *socolofi* highlights an additional challenge in India namely, illegal ornamental fish trafficking. The “Guidelines for import of ornamental fishes into India” [53] includes an “indicative list” of 92 exotic ornamental fish species that the Government of India has approved for import. Although *C. socolofi* is not on this list, it is widely available in Indian domestic trade ([67], this report).

Clearly, the fish were acquired illegally via international smuggling. Unfortunately, the “Guidelines” makes no clear or implicit declaration prohibiting the import of ornamental fish that are not on the “indicative list”, nor does it suggest that violators will face prosecution or even a fine. It simply states that “the import permit shall be cancelled forthwith and all the specimens imported destroyed without any notice to or permission of the importer”.

Given that the issue at hand involves not only fish trafficking but also the trafficking of accompanying (unidentified and often overlooked) parasites, merely cancelling import permits is a minor step toward protecting biodiversity and deterring traffickers. In reality, illegally imported exotic species are more likely to introduce parasites and diseases into the country because they bypass the import risk analysis and quarantine procedures of the importing country. Therefore, we recommend that the sale of a non-permitted ornamental fish species be treated as a criminal offence comparable to wildlife smuggling and implementing heavy penalties for this crime.
